# Early Insights From Clinical Trials of Typhoid Conjugate Vaccine

**DOI:** 10.1093/cid/ciaa370

**Published:** 2020-07-29

**Authors:** Kathleen M Neuzil, Buddha Basnyat, John D Clemens, Melita A Gordon, Priyanka D Patel, Andrew J Pollard, Mila Shakya, Firdausi Qadri

**Affiliations:** 1 University of Maryland School of Medicine, Baltimore, Maryland, USA; 2 Oxford University Clinical Research Unit, Patan Academy of Health Sciences, Kathmandu, Nepal; 3 Infectious Diseases Division, International Centre for Diarrhoeal Disease Research, Bangladesh (icddr,b), Dhaka, Bangladesh; 4 Malawi-Liverpool Wellcome Trust Clinical Research Programme, Blantyre, Malawi; 5 Institute of Infection and Global Health, University of Liverpool, Liverpool, United Kingdom; 6 Oxford Vaccine Group, Department of Paediatrics, University of Oxford, Oxford, United Kingdom; 7 National Institute for Health Research Oxford Biomedical Research Centre, Oxford, United Kingdom

**Keywords:** clinical trials, conjugate vaccine, typhoid, TyVAC

## Abstract

Clinical trials of typhoid conjugate vaccine (TCV) are ongoing in 4 countries. Early data confirm safety, tolerability, and immunogenicity of typhoid conjugate vaccine, and early efficacy results are promising. These data support World Health Organization recommendations and planned country introductions. Forthcoming trial data will continue to inform programmatic use of typhoid conjugate vaccine.

Typhoid fever is a systemic infection caused by *Salmonella enterica* serovar Typhi (*S*. Typhi). The relative importance of water in the transmission of typhoid has been recognized since the 19th century, before the causative agent of the disease was known. In the 20th century, many countries witnessed rapid declines in the incidence of typhoid fever following the introduction and improvement of water quality and sanitation systems [1–3].

While typhoid has been eliminated in much of the world, it continues to be a substantial public health threat in much of Asia, sub-Saharan Africa, and Oceania. Currently, typhoid fever disproportionately impacts children and marginalized populations. The burden of typhoid is likely underestimated due to difficulties in surveillance and diagnostic challenges. Current global estimates of typhoid fever are at least 11 million cases and > 116 000 deaths per year [4–6]. Complications arise in 10%–15% of untreated typhoid cases and include intestinal perforation, hemorrhage, and septic shock [7]. The case fatality rate of typhoid fever is 1%–4% with appropriate antibiotic and supportive treatment, and 10%–20% without such treatment [8].

Travelers to typhoid-endemic countries have long had access to vaccines. An older-generation, parenteral whole-cell vaccine was used extensively in the military. While the vaccine was efficacious, it was abandoned due to its reactogenicity profile [9]. Parenteral typhoid polysaccharide vaccine or an oral live attenuated vaccine has been used by travelers to endemic settings and for outbreak control. However, neither vaccine has been introduced into routine immunization programs. The broader use of these vaccines has been limited by the lack of a licensed formulation for children < 2 years of age, the need for multiple doses in the case of the live oral vaccine, a relatively modest level of protection, and limited duration of immunity.

In general, protein-polysaccharide conjugate vaccines have the advantages of improved immunogenicity in the youngest age groups and longer duration of immunity compared with unconjugated polysaccharide vaccines. A capsular polysaccharide of *S.* Typhi, the Vi antigen, bound to nontoxic recombinant *Pseudomonas aeruginosa* exotoxin A, was highly efficacious in Vietnamese children 2–5 years of age, achieving > 90% efficacy against the primary outcome of blood culture–confirmed symptomatic *S*. Typhi infection at 1 year [10]. Unfortunately, that vaccine has not yet made it to market. However, research and development of TCVs has continued. Nearly 20 years after the start of the trial in Vietnam, Typbar-TCV (Bharat Biotech International Ltd) became available on the global market and was prequalified by the World Health Organization (WHO) in 2017. This TCV has a Vi polysaccharide conjugated to a tetanus-toxoid protein carrier, and was first licensed in India in 2013.

In 2018, WHO released updated recommendations on the use of typhoid vaccines. Noting the continued high burden of typhoid fever and the alarming increase in antimicrobial resistance (AMR) in low- and middle-income countries, WHO recommended a single dose of TCV in typhoid-endemic countries for children 6 months of age and older, plus catch-up vaccination for children up to 15 years of age. The WHO further stated that decisions on the age of TCV administration, target population, and delivery strategy for routine and catch-up vaccination should be based on the local epidemiology of typhoid fever, including AMR patterns, and programmatic considerations of the routine childhood immunization program. The WHO recommended prioritization to countries with the highest burden of disease or a high burden of antimicrobial-resistant *S.* Typhi. The statement emphasized that data will be needed on coadministration of TCV and other routine childhood vaccines, and that countries should strengthen surveillance and monitor occurrence of AMR [7, 10]. Importantly, Gavi, the Vaccine Alliance (Gavi) opened a funding window for TCV support, and committed US$85 million to support TCVs in Gavi-eligible countries [11].

Although the WHO recommendation and Gavi financing decision are essential for new vaccine introduction in Gavi-eligible countries, they are not sufficient for all countries. Countries have many competing priorities for limited healthcare dollars. The WHO recommendations were based on safety and immunogenicity data and the demonstration of TCV efficacy in a typhoid challenge study performed among adults in the United Kingdom [12, 13]. Further information on typhoid vaccine effectiveness in endemic settings, and impact on important public health outcomes, could further inform country decision-making and drive country uptake.

The Typhoid Vaccine Acceleration Consortium (TyVAC) was launched in 2017 to accelerate the introduction of TCVs in low-resource countries. TyVAC has assembled a broad partnership of in-country and international organizations to generate necessary data on safety, immunogenicity, and impact of new TCVs, to facilitate decision-making at the regional and country level, and to ensure country readiness for vaccine introduction [14, 15]. Four TyVAC clinical trials are in progress (Table 1), designed to provide data in a variety of settings. Details of the trial design have been previously published [16–19].

**Table 1.** Summary of Typhoid Vaccine Acceleration Consortium (TyVAC) Clinical Trials (as of 12 December 2019)

Country	Design	Control Vaccine	Ages	Start Date	Duration	No. Vaccinated
Nepal	Individually randomized efficacy trial	Capsular group A meningococcal conjugate vaccine	9 mo–16 y	Nov 2017	2 y^a^	20 019
Malawi	Individually randomized efficacy trial	Capsular group A meningococcal conjugate vaccine	9 mo–12 y	Feb 2018	2–2.5 y^b^	28 142
Bangladesh	Cluster-randomized effectiveness trial	Live attenuated Japanese encephalitis	9 mo–16 y	Apr 2018	2 y	67 395
Burkina Faso	Individually randomized immunogenicity trial	Inactivated poliovirus vaccine	9–23 mo	Nov 2018	9 mo	250

^a^Case-driven interim efficacy.

^b^Case-driven design with a minimum of 2 years of follow-up.

While the trials are ongoing, TyVAC is committed to providing data to countries and policy-makers as early as feasible (Table 2). For example, safety data from > 100 000 participants in the TyVAC trials were presented in December 2018 to the Global Advisory Committee on Vaccine Safety (GACVS). The GACVS review also included data from early public-sector use of TCV in India and Pakistan (Figure 1). GACVS concluded that the “safety profile of the Typbar-TCV vaccine is reassuring, and no signals of serious adverse events [AEs] were presented.” GACVS recommends that countries introducing TCV into their routine immunization schedule or into campaigns make every effort to ensure robust monitoring of safety. This will provide additional data on coadministration of TCV with other routine childhood vaccines or in special populations, to detect any signals that require further investigation, and to maintain public confidence in the immunization program [20]. Investigational and programmatic introductions with TCV are shown in Figure 1.

**Table 2.** Data Gaps That Will Be Addressed by Typhoid Vaccine Acceleration Consortium (TyVAC)–Sponsored Trials

Data Elements	Status
Safety	•Bangladesh, Malawi, Nepal trial data—presented to WHO Global Advisory Committee on Vaccine Safety, 2018 •Data collection ongoing, including special populations (HIV-infected, HIV-exposed, malnourished children)
Immunogenicity in endemic settings outside of India	•Bangladesh, Malawi, Nepal trial data—presented at the 11th International Conference on Typhoid and Other Invasive Salmonelloses, 26–28 March 2019, Hanoi, Vietnam •Studies ongoing in Burkina Faso, Malawi, and Nepal to explore immunogenicity at 9 vs 15 months; with 1 vs 2 doses of vaccine; and in HIV-exposed children
Efficacy and impact in endemic setting	•Nepal—first-year efficacy data against laboratory-confirmed infection presented at the 11th International Conference on Typhoid and Other Invasive Salmonelloses, 26–28 March 2019, Hanoi, Vietnam and in peer-reviewed literature. Trial ongoing. •Individually randomized trial ongoing in Malawi •Cluster-randomized trial followed by catch-up vaccinations ongoing in Bangladesh
Coadministration with other vaccines	•Burkina Faso: TCV with measles-rubella, yellow fever, meningitis A •Malawi: TCV with measles-rubella
Duration of protection	•Trials currently funded for 2–2.5 y of follow-up

Abbreviations: HIV, human immunodeficiency virus; TCV, typhoid conjugate vaccine; WHO, World Health Organization.

**Figure 1.**
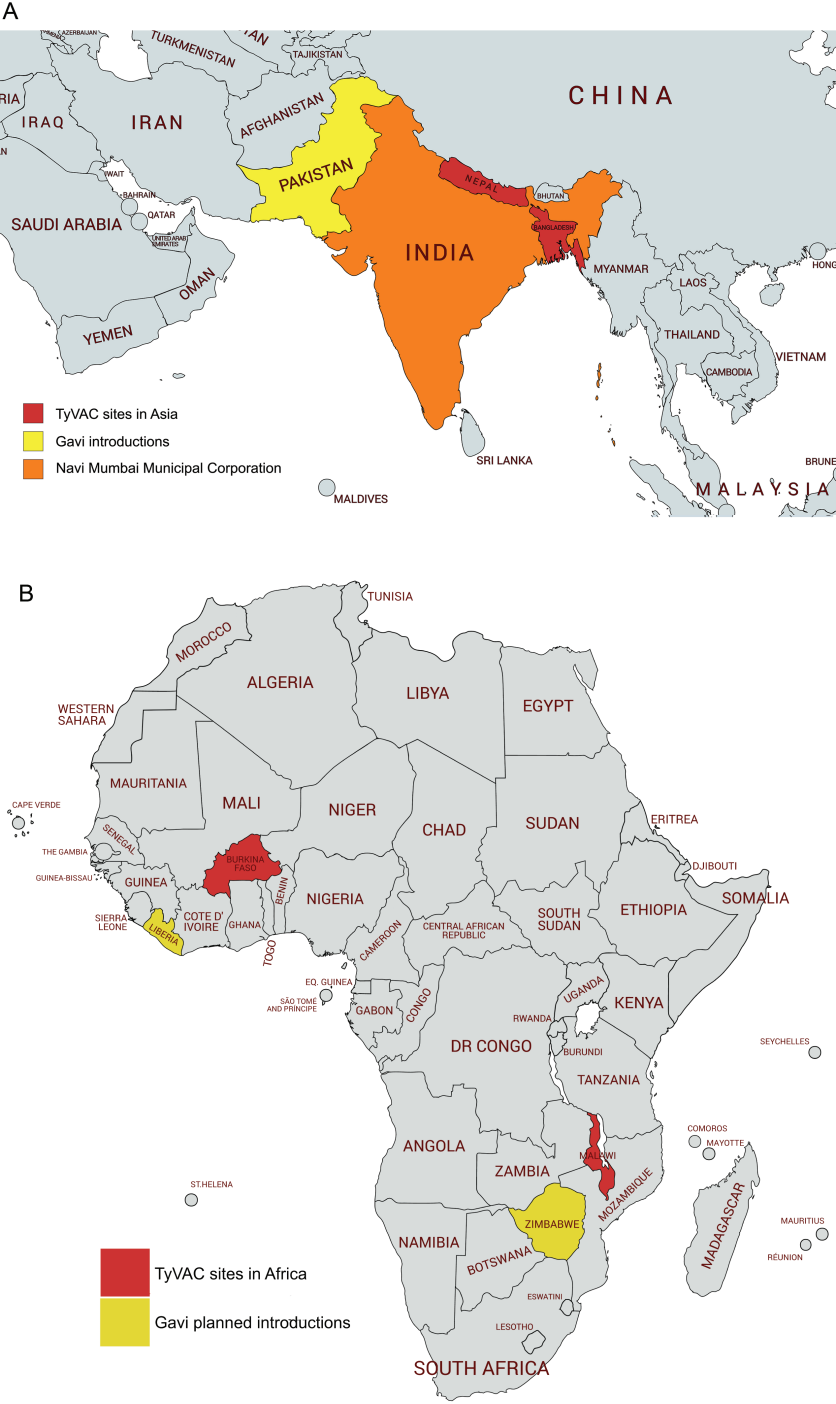
Trials and programmatic introductions of typhoid conjugate vaccine in Asia (*A*) and Africa (*B*), as of December 2019. Abbreviation: DR Congo, Democratic Republic of Congo; TyVAC, Typhoid Vaccine Acceleration Consortium.

At the 11th International Conference on Typhoid and Other Invasive Salmonelloses, TyVAC data on safety and immunogenicity from the Bangladesh, Malawi, and Nepal trials, and interim efficacy data from Nepal, were presented. While the safety results remain blinded, serious AEs represented the pattern of typical childhood diseases seen in these settings. In Malawi, a subset of approximately 600 children was actively followed, and mild fever and irritability were the most common AEs in the week following vaccination with either TCV or the control vaccine, capsular group A meningococcal conjugate vaccine (MenA). In the Bangladesh cluster-randomized trial, which used the live attenuated Japanese encephalitis vaccine as the control, > 4800 children had AEs assessed at 1 week following vaccination. Mild fever was the most common AE in both vaccine groups. Similarly, in Nepal, fever and injection site pain were the most common AEs 1 week following vaccination with TCV or MenA.

The immunogenicity subset consisted of approximately 1500, 600, and 1300 participants in Bangladesh, Malawi, and Nepal, respectively. Based on Vi antibody response 1 month following vaccination, robust immunogenicity was observed at all sites and in all age groups. These results are comparable to those that served as the basis of licensure in India [13]. Longer-term immunogenicity sampling is ongoing.

For the randomized controlled efficacy trial in Nepal, the primary endpoint was blood culture–confirmed typhoid fever. A total of 10 005 participants received TCV and 10 014 received MenA. An interim analysis was conducted after a prespecified minimum of 45 cases was reached, after approximately 1 year of follow-up. The incidence of typhoid fever was 428 per 100 000 in the MenA control group, confirming the high burden of disease in children in this setting. Blood culture–confirmed typhoid fever occurred in 7 participants who received TCV and 38 receiving MenA for a vaccine efficacy of 81.6% (95% confidence interval, 58.8%–91.8%; *P* < .001). The point estimate for efficacy was higher when the standard WHO definition of typhoid fever—requiring 3 days of fever—was used [21, 22]. The Nepal trial will continue through 2 years of follow-up for all participants, at which time analyses of efficacy in subgroups and against important public health outcomes, such as hospitalizations and typhoid complications, will be conducted.

While *S*. Typhi continues to be a substantial public health threat in low-resource settings, the tremendous progress and global momentum toward typhoid control were evident at the 11th International Conference on Typhoid and Other Invasive Salmonelloses. Early data from the TyVAC trials presented at the conference confirm the safety, tolerability, and immunogenicity of Typbar-TCV. Likewise, the 1-year efficacy results from Nepal are promising. These data further support the WHO recommendations and the decision by several countries to introduce TCV (Figure 1). The TyVAC trials are ongoing and will generate additional data on safety, immunogenicity, and efficacy (including indirect effects and against multiple clinical outcomes) in diverse populations. While the promise of such data should not delay country decision-making, countries have different interests, readiness, and capacities to support introduction. Forthcoming data will continue to inform additional country uptake and programmatic use of TCV.

## Notes

***Acknowledgments.*** The authors acknowledge the support of Leslie Jamka for her editorial assistance; TyVAC’s partners; our data safety and monitoring board members; and all of the children and families for their participation.

***Financial support.*** This work was supported by research funding from the Bill & Melinda Gates Foundation (grant number OPP1151153).

***Supplement sponsorship.*** This supplement is funded with support from the Coalition against Typhoid Secretariat, housed at the Sabin Vaccine Institute in Washington, DC and made possible by a grant from the Bill & Melinda Gates Foundation.

***Potential conflicts of interest.*** A. J. P. is Chair of the UK DHSC JCVI and the European Medicines Agency scientific advisory group on vaccines; is a member of the WHO’s Strategic Advisory Group of Experts on Immunization (SAGE); and is a National Institute for Health Research Senior Investigator. K. M. N. is a member of the WHO’s SAGE. All other authors report no potential conflicts. All authors have submitted the ICMJE Form for Disclosure of Potential Conflicts of Interest. Conflicts that the editors consider relevant to the content of the manuscript have been disclosed.
